# The pentagram of concussion: an observational analysis that describes five overt indicators of head trauma

**DOI:** 10.1186/s13102-022-00430-4

**Published:** 2022-03-15

**Authors:** Joshua A. Beitchman, Brendan A. Burg, Dylan M. Sabb, Ario H. Hosseini, Jonathan Lifshitz

**Affiliations:** 1 BARROW Neurological Institute at Phoenix Children’s Hospital, Phoenix, AZ USA; 2 Child Health, University of Arizona College of Medicine - Phoenix, Phoenix, AZ USA; 3 Joe R. and Theresa Lozano Long School of Medicine, University of Texas Health Science Center at San Antonio, San Antonio, TX USA; 4 Phoenix VA Health Care System, Phoenix, AZ USA; 5 Family and Community Medicine, Davis Hospital, University of California, CA Sacramento, USA; 6 Private Practice, Bellingham, WA 98229 USA; 7 UT Southwestern Medical Center, Children’s Medical Center Dallas, Dallas, TX 75235 USA; 8 University of Southern California, Los Angeles, CA 90089 USA; 9 University of California Davis, Sacramento, CA 95817 USA; 10 Neurotrauma & Social Impact research team, University of Arizona College of Medicine - Phoenix, 7th Floor BSPB | 475 N. 5th St., Phoenix, AZ 85004-2127 USA

**Keywords:** Brain injury, Fencing response, Concussive convulsion

## Abstract

**Background:**

Multifarious clinical presentations of traumatic brain injury (TBI) makes detection difficult. Acceptance of the Fencing Response as an indicator of moderate TBI with localization to the brainstem expanded interest towards other possible indicators.

**Methods:**

We hypothesized that an individual experiencing traumatic forces to the head resulting in concussion could display additional brainstem-mediated responses. Using YouTube™, videos were systematically evaluated for mechanical forces imposed on the head with a subsequent, observable behavior. Searches identified 9.9 million non-unique videos in which 0.01% were viewed and 79 met inclusion criteria. Videos of head injuries occurred during athletic activity (57%), assaults (38%), automobile accidents (4%) and impact by an inanimate object (1%).

**Results:**

Individuals with acute head injury were identified as adults (70%; n = 55), teens (29%; n = 23), and children (1.2%; n = 1). Those identified as males made up majority of injured persons (n = 77♂, 2♀). Individuals in the videos were observed to demonstrate the Fencing Response (47%; n = 37), seizing (44%; n = 35), snoring (24%; n = 19), crying (7.6%; n = 6), and vomiting (3.8%; n = 3).

**Conclusion:**

Each response, which together comprise the “Pentagram of Concussion”, indicates the presence of traumatic forces to the head that present with one or more pentagram signs that would localize dysfunction to the brainstem. Clinical consideration of these responses helps to immediately identify patients at high risk for a brain injury with brainstem involvement that may have otherwise been mistaken for a different diagnosis.

## Introduction

Traumatic brain injuries (TBI) are unique, where each one results in patient-specific clinical signs and symptoms. Sideline and initial clinic evaluations of TBI assess neurological function in the following domains: cognitive (orientation, memory, concentration), motor (balance problems, dizziness), visual (blurry vision, sensitivity to light), behavioral (changes to sleep, sensitivity to noise, headaches, fatigue drowsiness), and affective (nervousness, anxiety, depression, irritability). However, these patient-specific identifiers typically present hours to days following TBI, and so less useful for on-field indication of injury that requires medical attention.

An acutely brain-injured individual may act uncharacteristically, causing naïve bystanders to become perplexed or frightened. Involuntary, observable actions subsequent to TBI are the product of transient neurological dysfunction induced by the summed biomechanical forces applied to the brain [1]. These signs and symptoms of brain injury commonly overlap with other psychological, neurological or musculoskeletal conditions, which complicates the differential diagnosis [2]. The development of neurocognitive assessments has helped add objectivity to the diagnostic process. Yet, these assessments are laborious, require specialists to administer, are not able to definitively diagnose concussion alone, and are not practical at the scene of the incident [3]. Thus, most mild (*i.e.* concussions) and moderate TBIs are diagnosed clinically and not by laboratory tests or imaging (CT, MR) studies. The combination of clinical subjectivity and imperfect evaluation inevitably results in the misdiagnosis of brain injury [4, 5]. Unidentified, brain-injured patients may resume normal activity, increasing their risk for a delayed recovery and the development of more severe post-concussive symptoms. As a result, daily aspects of their personal and professional lives become significantly impaired [6–8].

Observable indicators of TBI improve detection, allow for rapid initiation of clinical management, and justify conservative return to activity guidelines. Hosseini and Lifshitz first reported the Fencing Response as an overt, visual indicator of moderate TBI in 2009 [9]. Over the past decade, identification of the Fencing Response through videos shared on social media (i.e. Twitter™) has initiated a discussion amongst health care professionals and lay individuals who have a vested interest in identifying brain-injured individuals. Presentation of the Fencing Response has been attributed to acute neurological dysfunction in the lateral vestibular nucleus (LVN) in the brainstem. Mechanical damage in this location likely activates motor neurons that sustain forearm and leg posturing. Similar dysfunction to adjacent, vulnerable nuclei likely occurs, eliciting additional indicators of brain injury. This communication serves to investigate the presentation of brainstem-mediated responses that occur in response to mechanical (concussive) forces to the head. Identification of these additional, observable indicators of brain injury aids health care providers and responsible parties to identify acute TBI and initiate treatment regimen.

### Methods

A systematic analysis of videos in the public domain was conducted between June-July 2017 to identify individuals who received a mechanical force to the head and exhibited a subsequent, observable response. Videos uploaded to the online YouTube™ site (Google Inc, San Bruno, CA; http://www.youtube.com) were used as the primary source of data. The study used publicly available videos to record observations; investigators did not access, analyze, or create private or protected health information.

Individual consent from personal accounts or patrons displayed in the videos was unwarranted as the videos fall under the ‘fair use’ and ‘creative commons’ provisions of YouTube™. Each factual video was uploaded by an individual and videos were used for non-profit educational purposes. Furthermore, as the individuals who uploaded the videos have no profitable share in the video, the use of these videos fell under all four principles of the fair use guidelines outlined by YouTube™ and should not be in violation of their policy or copyright. The creative commons license of YouTube™ also grants a third party permission to use their work when uploading a video. By indicating a creative common’s license, the video creators grant the entire YouTube™ community the rights to reuse the video.

**Fig. 1:**
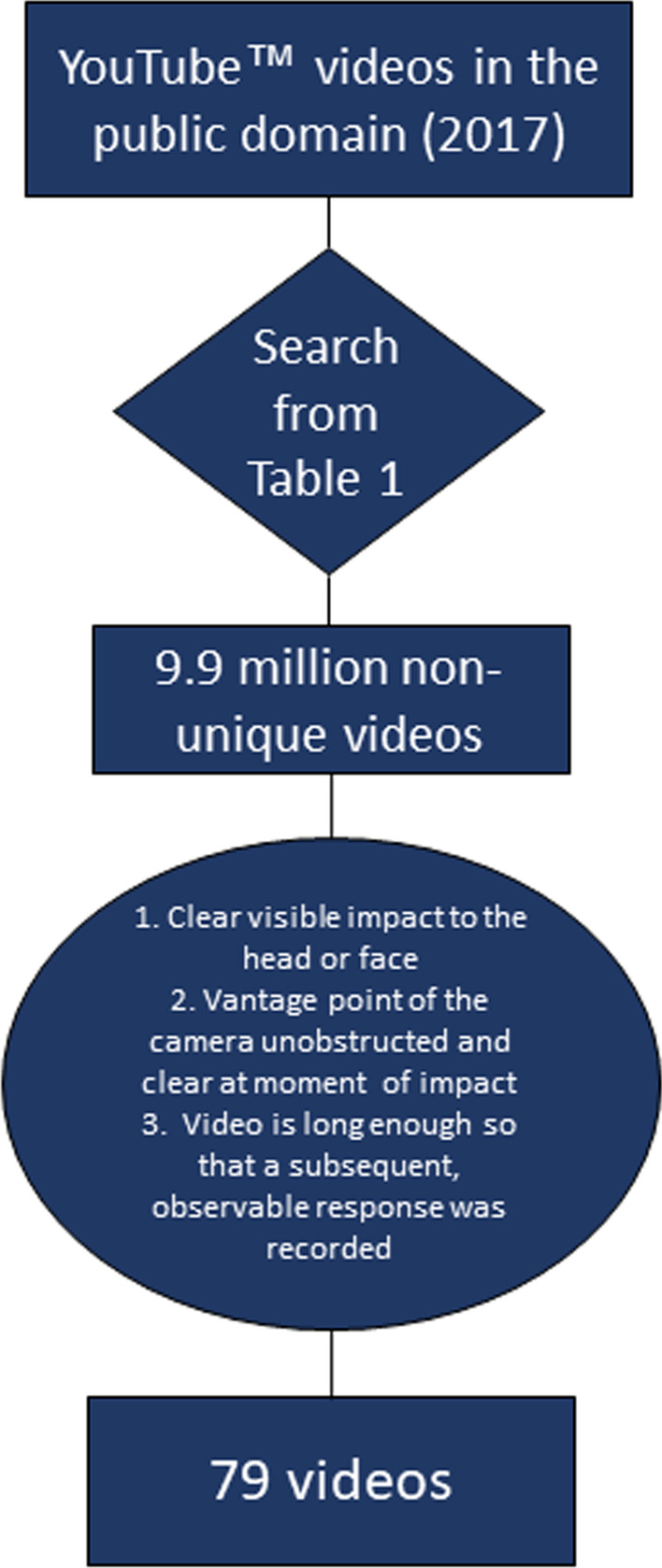
Flow diagram for selection criteria of videos in the public domain to the 79 videos analyzed

Search results were the product of reviewers using a combination of terms such as “concussion”, “knocked out”, “seizure”, “snoring”, “crying”, and “unconscious” as shown in Table 1. Combinations of terms first queried general head injury and knock out videos. Once overt signs of concussion were identified, additional queries with specific symptoms were made to collect relevant videos for analysis. Searches resulted in 9.9 million non-unique videos and were sorted online based on relevance. To be considered for inclusion in the analysis, the uploaded video must have met the following criteria: (1) clear visible impact to the head or face; (2) the vantage point of the camera is unobstructed and clear at the moment of impact; (3) the video is long enough so that a subsequent, observable response was captured on video. These criteria allowed for the screening of approximately 1000 videos in which a total of 79 unique videos met criteria (Fig. 1). Any duplicate videos under different titles (e.g. compilation or commentary) showing the same footage were disregarded. For each video, the initial reviewers determined the type of event (Table 2), age group of the individual injured (based on physical characteristics; adult 18+; teen 13–18; child < 13) (Table 2), unresponsiveness of the injured individual as demonstrated by video (Table 2), binary gender designation (Table 2), impact direction, and limb laterality of response (involvement of upper and/or lower extremities). Each of the 79 videos meeting inclusion criteria were then independently reviewed by a second reviewer for the same characteristics. In the event that the second reviewer categorized the video different from the first reviewers, a third independent reviewer broke all ties. All data analysis was collected and performed in a customized Microsoft Office Excel spreadsheet. Data were evaluated for contributing variables on the basis of age and gender to determine if certain responses were more likely to occur in these demographics.

**Table 1:** YouTube™ search terms and results

Website	Search term	Total videos queried	Percentage viewed (%)
YouTube™	Knocked unconscious sports	1,640,000	0.01
YouTube™	Knocked the f*** out	1,390,000	0.01
YouTube™	Football knockout	620,000	0.01
YouTube™	Brutal boxing knockouts	599,000	0.02
YouTube™	Knocked out cold	597,000	0.02
YouTube™	Weird reaction after knockout	279,000	0.04
YouTube™	Skateboard head slam	27,200	0.37
YouTube™	Skateboard concussion	8160	1.23
YouTube™	Seizure after getting hit in the head	75,500	0.13
YouTube™	Seizure after concussion	11,800	0.85
YouTube™	Seizure after knockout	7630	1.31
YouTube™	Head injury convulsion	3840	0.49
YouTube™	KO seizures	3190	1.25
YouTube™	UFC knockout seizure	2410	4.15
YouTube™	Snoring after getting knocked out	21,600	0.37
YouTube™	Vomit after concussion	17,000	0.59
Y	Crying after getting knocked out	4,690,000	0.002
	**Total**	**9,993,330**	**0.01**

Search terms used to query YouTube™ videos that returned non-zero search results. Percentage of videos reviewed for each result shown in the last column. Search terms are grouped by general terms meant to capture all potential videos related to the topic, followed by search terms specifically related to components of the Pentagram of Concussion. Search terms are sorted by total videos queried

**Table 2:** Distribution of observed head injuries by type, age, unresponsiveness following impact, and gender

*Types of events*	
Assault	38%
Athletic	57%
Automobile	4%
Impact	1%
**Total**	**100%**
*Age group*	
Adult	55
Teen	23
Child	1
**Total**	**79**
*Unresponsiveness*	
N/A	1.3%
No	34%
Yes	65%
**Total**	**100%**
*Gender*	
Female	2
Male	77
**Total**	**79**

Distribution of observed head injuries by type of event, age group, unresponsiveness following impact, and gender

## Results

During the 2-month analysis period, which included all video uploaded to date, YouTube™ was queried for videos that demonstrated an individual who received an impact to the head and exhibited a subsequent, observable response. Using the searches listed in Table 1 and inclusion parameters described previously, 79 videos met criteria for analysis. Demographic characterization of these videos identified injuries occurred during athletic activity (57%), assaults (38%), automobile accidents (4%) and impact by an inanimate object (1%) (Table 2). Adults were primarily injured, representing 70% (n = 55) of the analyzed videos, followed by teens (29%; n = 23) and children (1.2%; n = 1) (Table 2). Individual who were identified as males made up majority of injured persons (n = 77♂, 2♀) (Table 2).

**Fig. 2:**
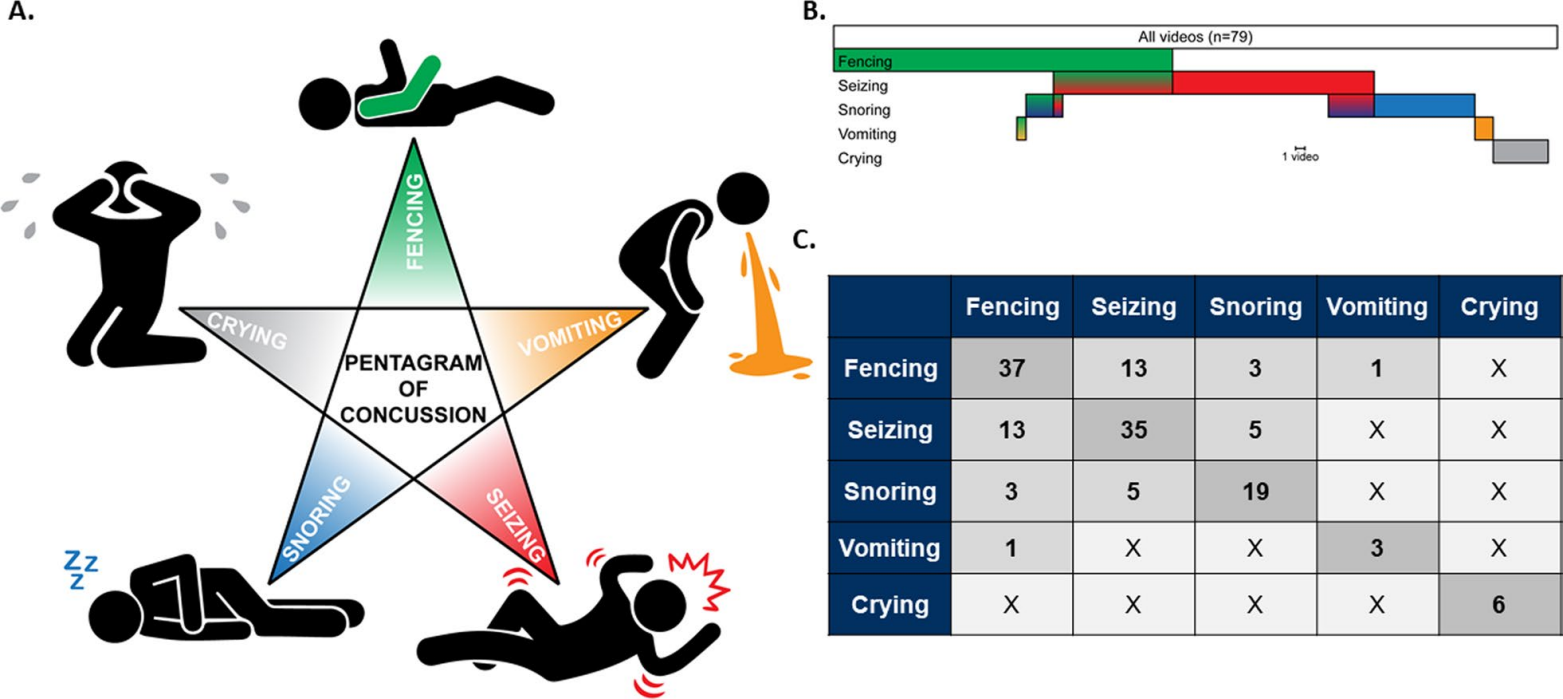
**A** Pentagram of Concussion depicting each pennant as the Fencing Response, seizures, snoring, vomiting, and crying. **B** Color bars indicate the distribution of each observable response identified immediately following head injury as a fraction of all videos. Bar length is proportional to the number of videos exhibiting each response. More than one color in a single bar indicate the occurrence of multiple responses following a single head injury. **C** Number of videos viewed with each observable response following head injury. Total videos identifying a specific response are shown with dark grey background

**Fig. 3:**
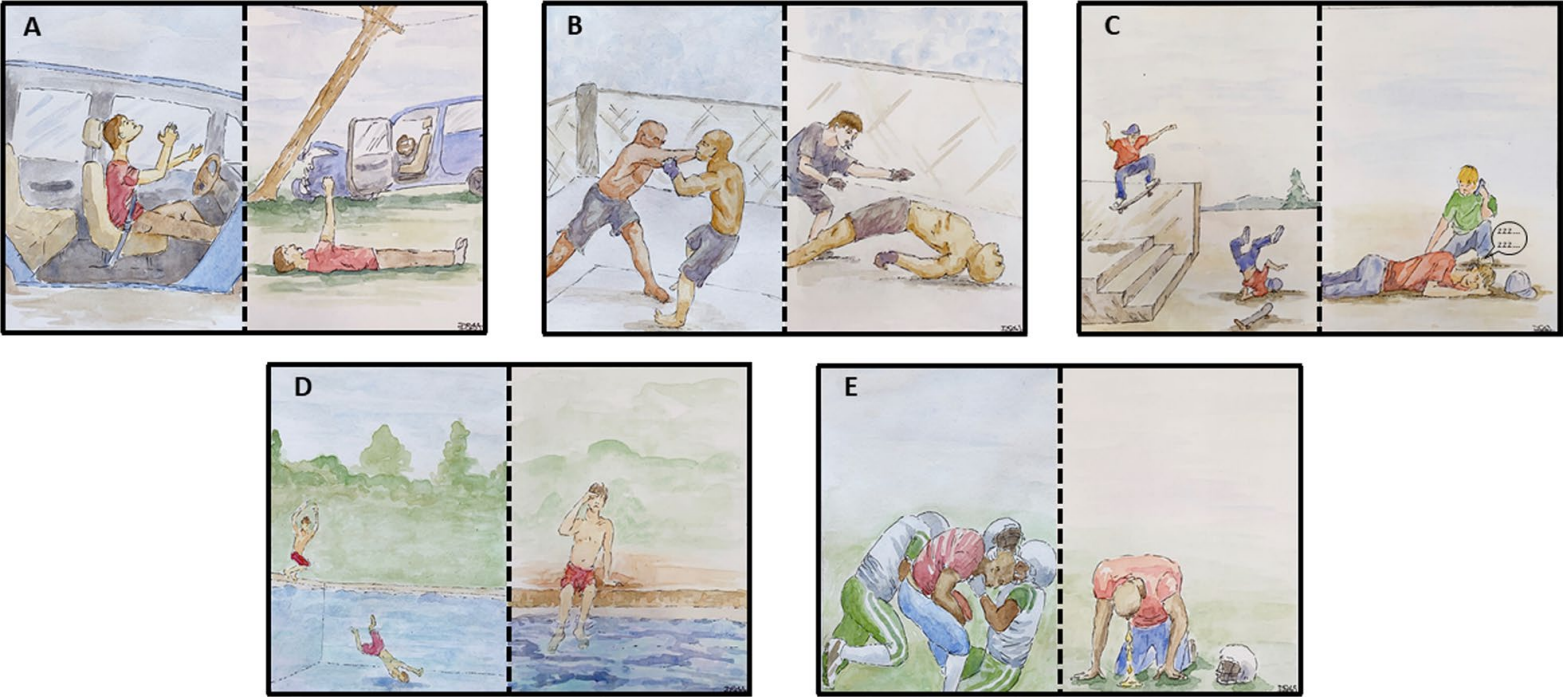
Artistic illustrations of each response in the Pentagram of Concussion. Each two-panel story depicts commonly observed responses to head injury from the videos analyzed. In each scenario, an individual receives a traumatic impact to the head and then an observed response indicative of brain injury. Each aspect of the Pentagram of Concussion is shown. **A** Fencing response, **B** seizures, **C** snoring, **D** crying, **E** vomiting. While shown in isolation here, it should be recognized that more than one response may occur simultaneously or in succession of one another

Of the videos analyzed, 65% (n = 51) of subjects were found to be unresponsive after experiencing head trauma (Table 2). Unresponsiveness was defined as lack of self-initiated movement or response to physical or auditory stimulation from the injured individual. The Fencing Response was the most prevalent observed response following injury (47%; n = 37), followed by seizures (44%; n = 35), snoring (24%; n = 19), crying (7.6%; n = 6), and vomiting (3.8%; n = 3) (Fig. 2B, C) where more than one response may be present in a video. Together, these five responses are coined “The Pentagram of Concussion”, with each one representing a pennant (Fig. 2A). Artistic illustration of each of these responses was constructed based on the videos analyzed (Fig. 3A: Fencing Response, Fig. 3B: Seizing, Fig. 3C: Snoring, Fig. 3D: Crying, Fig. 3E: Vomiting). Each response was found to occur subsequent to multiple injury mechanisms and those illustrated are for demonstration purposes only. Observed responses to injury were not mutually exclusive. The Fencing Response was found to coincide with seizing (n = 13), snoring (n = 3), and vomiting (n = 1), while seizing was found to coincide with snoring (n = 5). Crying was not found to coincide with any of the observed responses in the videos analyzed (Fig. 2B, C). The side of impact did not determine the laterality of the fencing response (*i.e.* left-sided impact leads to Fencing Response in Left Upper Limb) or seizures. Finally, analysis on the basis of age indicated that 57% of adults exhibited the Fencing Response while 78% of teens exhibited a seizure.

## Discussion

Our aim was to identify observable, individual responses that indicate an individual had experienced traumatic forces to the head severe enough to cause a brain injury. These data show that application of traumatic forces to the head result in five possible responses. Through the collation of 79 videos by selective inclusion criteria, we observed the involuntary demonstration of the Fencing Response, seizures, snoring, crying, and vomiting after a head injury. Responses are not mutually exclusive as simultaneous or subsequent occurrences were observed in 22 videos. Moderate diffuse brain injuries disrupt the blood–brain barrier (BBB) and injure neurons in the brainstem which elicits the Fencing Response. We suggest that application of traumatic forces to the head results in dysfunction to adjacent vulnerable brainstem nuclei and mediate each additional response studied here. By considering each response described in the “Pentagram of Concussion”, patients who are at high risk for being brain-injured can be immediately identified and not otherwise mistaken for a different diagnosis.

The identification of brain injuries through observed responses rather than labyrinthine assessments allows for more timely evaluation and initiation of patient management. Observed responses to a brain injury are the result of transient neurological dysfunction from the sum biomechanical forces that the individual’s brain experienced [1]. The majority of our videos (65%) demonstrated a lack of responsiveness following injury. As brain injuries may occur without loss of consciousness, we broaden our inclusion criteria from our previous study to identify additional overt indicators of brain injury. Furthermore, case-reports of individuals who have experienced the Fencing Response communicate awareness of their actions despite an inability to control them, which indicates that one may only appear unconscious. Acting as a fulcrum at the cephalic flexure, the brainstem is uniquely vulnerable to neuronal stretching and BBB disruption with the application of brain injury forces as the cerebrum is displaced inside the skull. It is thus reasonable that brainstem-mediated actions, not necessarily reflexes, may occur with TBI.

Identification of the Fencing Response provided the first studied observable indicator of TBI that provides insight into the magnitude of injury-related forces. Since our initial publication in 2009, the Fencing Response has gained acceptance, with inclusion into the National Football League and Berlin Concussion statements, in addition to a 1431% increase in Google searches (2009–2018 compared to 2004–2009). In studies evaluating sports related concussions (SRC), Tényi *el al.* and McCrory et al*.* have independently observed the Fencing Response in 16 and 25 cases respectively [10, 11]. In our initial characterization, we provide histopathological evidence that the Fencing Response coincides with non-focal BBB disruption and neuronal injury in the LVN acutely after moderate, but not mild TBI in the rat [9]. Neurovascular compromise and stretching of the cerebellar peduncles result in activation of the ipsilateral LVN and production of the Fencing Response. Directionality of forces may correlate with laterality of the Fencing Response as Tényi *el al.* describes a significant relationship between the patient’s head turning towards the extended arm and directionality of forces [10]. The absence of this finding here and in our 2009 publication may be due to variations in aggregate force vectors of brain injury or limited sample populations. Nonetheless, with either or both arms, demonstration of the Fencing Response necessitates medical attention, and is indicative of a moderate brain injury with involvement of the brainstem.

Gyrations following concussions have been described as injury-induced convulsions, “tonic posturing” and “impact seizures” [10–13]. There is considerable debate on the neuronal origination of these events. McCrory postulates that the observed convulsions are due to a transient decerebration, while Tényi states that the latency and nature of convulsions suggests a cortically-mediated event [10, 12]. Impact reconstruction of American football players who received concussions allowed for fine element modeling to evaluate for changes to strain and deformation of brain tissue that the patients likely experienced. The modeling indicated that brain-injured patients who convulsed as a result of traumatic forces to the head resulting in concussion experienced a lower magnitude of strain and deformation of cerebral white matter compared to those who lost consciousness [14]. Cournoyer et al*.* interprets these data to indicate that convulsing patients experienced less neuronal injury, thus implying that the preservation of neuronal function in certain parts of the brain and dysfunction in others allows for muscle groups to be activated and manifest as a seizure.

We postulate that activation of the brainstem reticular formation and pontine tegmentum by transient neuronal dysfunction induced by traumatic forces to the head resulting in concussion is responsible for seizures after injury. Direct electrostimulation of the reticular formation has been shown to produce desynchronized convulsive attacks compared to the hypersynchronous cortically-mediated convulsions [12, 15, 16]. Furthermore, independent operation of neuronal circuits may produce clinically distinct convulsions as activation of the reticular formation has been shown to supplant an ongoing cortical seizure [16]. Demonstration of either a tonic or clonic component during a convulsion may be dependent on the magnitude of the stimulating current applied to the brainstem reticular formation whereas full activation leads to tonic movements and partial activation leads to a specific type of clonus [15]. Ultimately, additional investigation is needed to decipher if TBI-induced convulsions are the result of cortical or brainstem injury, however the immediacy of the concern may not alter health care delivery. Certain factors such as latency to onset, duration, and observed posturing may help identify injuries with localization to the brainstem or cortex as suggested by Tényi et al*. *[10]. The age of the injured individual may also be important as convulsions represented the most likely response to occur in teens. This finding may indicate a predisposition of the adolescent brain to seizures due to immaturity and incomplete myelination compared to the mature adult central nervous system or an artifact of a limited sample size.

Vocalization has been identified rarely in the presence of convulsions [10]. We observed nineteen instances of snoring, and only five that coincided with seizures. While it is possible that these events represent an ictal cry, eleven episodes occurred independently of seizures and three instances coincided with the Fencing Response, which reduces the likelihood of it being such. Of note, in all nineteen instances of snoring, the injured individual was determined to be unresponsive. Thus, these vocalizations may represent a brief apneustic breathing episode from injury to the apneustic center and pons in the mesencephalon. Alternatively, these vocalizations may also be due to obstructive oropharyngeal breathing from intermittent activation/relaxation from the oropharyngeal muscles following injury to the nucleus ambiguous.

Vomiting is another physiologically complex reflex requiring coordination of multiple nuclei, including the area postrema, dorsal motor nucleus of the vagus nerve, and the ventral respiratory nucleus. Vomiting as a response to TBI may indicate pathophysiological activation of one or multiple of these brainstem regions.

Finally, the observed crying response after brain injury initiated quickly and continued uncontrollably. We suggest injury-induced crying as a brainstem-mediated response rather than a cognitively-mediated (limbic) event. Transient neurological dysfunction in the superior salivatory nucleus could produce crying and cannot be dismissed as an indicator of brain injury.

To the best of our knowledge, we are the first to systematically evaluate the presence of snoring, vomiting, or crying following brain injury despite their acceptance as indicators for concussion evaluation [17–19]. We suggest that each of these other four pennants of the Pentagram of Concussion has an associated, vulnerable region in the brainstem, as summarized above and in Table 3. As with the Fencing Response and seizures, each of these responses has anatomically vulnerable brainstem nuclei that are susceptible to transient neurological dysfunction. Observing any of the five responses included in the Pentagram of Concussion would broaden a differential diagnosis of brain injury to one that includes damage to the brainstem. However, future studies will need to determine the extent of injury to these nuclei and whether any single pennant of the Pentagram of Concussion differentiates injury severity.

**Table 3:** Pentagram of Concussion Hypothesized Anatomical Mechanisms

Response	Nuclei	Tract	Effector
*Fencing response*			
First order	Lateral vestibular nucleus	Vestibulospinal fasciculus	Ipsilateral limb extensor (excitatory),
﻿ Second order	N/A	N/A	Ipsilateral limb flexors (inhibitory)
*Seizing*			
﻿ First order	Oral pontine reticular nucleus	Reticulospinal	Proximal muscles, axial muscles
﻿ Second order	N/A	N/A	
*Seizing*			
﻿ First order	Pontine tegmentum	Reticulospinal	Proximal muscles, axial muscles
﻿ Second order	N/A	N/A	
*Snoring*			
﻿ First order	Nucleus ambiguous	Cranial nerve IX, X	Oropharyngeal musculature
﻿ Second order	N/A	N/A	
*Vomiting*			
﻿ First order	Area postrema	Unnamed efferent tracts	Cardiac sphincter, intercostal
﻿ Second order	Vomiting center; nucleus tractus solitarii	Dorsal motor nucleus of X, ventral respiratory nucleus	Motor neurons, abdominal motor neurons
*Vomiting*			
﻿ First order	Dorsal motor nucleus of cranial nerve X	Cranial nerve X	Cardiac sphincter
﻿ Second order	N/A	N/A	
*Vomiting*			
﻿ First order	Ventral respiratory nucleus	Reticulospinal, phrenic nerve	Intercostal motor neurons, abdominal
﻿ Second order	N/A	N/A	Motor neurons
*Crying*			
﻿ First order	Superior salivatory nucleus	Greater petrosal nerve	Lacrimal gland
﻿ Second order	Pterygopalatine ganglion	V1	

Each pennant of the Pentagram of Concussion may be attributed to transient neurological dysfunction of a brainstem nuclei following head injury. This table represents the proposed mechanisms of each response. Each nucleus has neuroanatomical vulnerability due to the anatomical location and known diffuse BBB disruption in the brainstem. Activation of downstream neuronal circuitry (first and second order nuclei) can activate an effector producing the observed response

Inherent limitations exist in this report. Our study data are biased by the videos uploaded by individuals to YouTube™. While each response may be more prevalent amongst the general TBI population, we were limited to those in the public domain and returned in the search results. Searches (listed in Table 1) were initially broad and narrowed based on observed responses. In doing so, the inclusion of the specific components of the Pentagram appeared in search terms. This limits the interpretation of the incidence for each of the responses, however, the extent of the video database of YouTube makes analyzing every uploaded video impractical. Further, without live evaluation of the injured individual, loss of consciousness or other aspects of injury severity (complicated mild to moderate TBI, other co-morbid injuries) could not be determined, including acute and long-term follow-up. The low incidence of concussion in sport at a single site or facility makes long-term follow-up a challenge. Ongoing or future multi-center studies, such as the NCAA-DOD CARE consortium, may provide insight into long-term outcomes. By the nature of the videos available, determination of the magnitude of forces was not possible. Furthermore, a significant gender bias towards individuals identified as males was noted. While it is suspected that these data hold for all genders, future studies can determine the specificity and sensitivity of each pennant of the Pentagram of Concussion for gender, age, and other demographic variables. Finally, our characterization of age could be biased by physical determinants. Despite these limitations, the videos analyzed and presented here demonstrate evidence for expansion of the observable responses stemming from concussion.

## Conclusions

Detection of concussions is difficult at best due to their multiple clinical presentations. Current guidelines continue to use non-objective language when providing clinicians with identifiers of brain-injured patients. Objective measures are needed in order to increase the confidence in the clinical diagnosis of concussions. The proposed Pentagram of Concussion provides five overt, visual indicators that rapidly aid in the identification of brainstem involvement following an injury to the head. Inclusion of these responses into the existing criteria to detect TBI can aid diagnosis without compromising other testing. Furthermore, these responses can be applied by non-specialists in environments that lack concussion-trained experts. Finally, in accord with current consensus statements, these metrics may be readily implemented in initial or video review of SRC.(2, 13) As such, we propose that the responses in the Pentagram of Concussion should be used by clinicians in combination with additional evidence-based assessments in their evaluation of concussion. By doing so, brain-injured patients may be identified more readily following head injury and receive appropriate treatments and interventions that aid in their recovery.

## Data Availability

The datasets used and analyzed during this study are available from the corresponding author upon reasonable request.

## References

[1] Lovell M (2009). The management of sports-related concussion: current status and future trends. Clin Sports Med.

[2] Patricios JS, Ardern CL, Hislop MD, Aubry M, Bloomfield P, Broderick C (2018). Implementation of the 2017 Berlin Concussion in Sport Group Consensus Statement in contact and collision sports: a joint position statement from 11 national and international sports organisations. Br J Sports Med.

[3] Dessy AM, Yuk FJ, Maniya AY, Gometz A, Rasouli JJ, Lovell MR (2017). Review of assessment scales for diagnosing and monitoring sports-related concussion. Cureus..

[4] McCrea M, Guskiewicz K, Randolph C, Barr WB, Hammeke TA, Marshall SW, et al. Effects of a symptom-free waiting period on clinical outcome and risk of reinjury after sport-related concussion. Neurosurgery. 2009;65(5):876–82; discussion 82–3. 10.1227/01.NEU.0000350155.89800.00 19834399

[5] Guskiewicz KM, McCrea M, Marshall SW, Cantu RC, Randolph C, Barr W (2003). Cumulative effects associated with recurrent concussion in collegiate football players: the NCAA Concussion Study. JAMA.

[6] Asken BM, McCrea MA, Clugston JR, Snyder AR, Houck ZM, Bauer RM (2016). "Playing Through It": delayed reporting and removal from athletic activity after concussion predicts prolonged recovery. J Athl Train.

[7] Elbin RJ, Sufrinko A, Schatz P, French J, Henry L, Burkhart S, et al. Removal from play after concussion and recovery time. Pediatrics. 2016;138(3). 10.1542/peds.2016-0910 PMC5005026 27573089

[8] McAllister TW (1992). Neuropsychiatric sequelae of head injuries. Psychiatr Clin North Am.

[9] Hosseini AH, Lifshitz J (2009). Brain injury forces of moderate magnitude elicit the fencing response. Med Sci Sports Exerc.

[10] Tenyi D, Gyimesi C, Horvath R, Kovacs N, Abraham H, Darnai G (2016). Concussive convulsions: A YouTube video analysis. Epilepsia.

[11] McCrory PR, Berkovic SF (2000). Video analysis of acute motor and convulsive manifestations in sport-related concussion. Neurology.

[12] McCrory PR, Bladin PF, Berkovic SF (1997). Retrospective study of concussive convulsions in elite Australian rules and rugby league footballers: phenomenology, aetiology, and outcome. BMJ.

[13] McCrory P, Meeuwisse W, Dvorak J, Aubry M, Bailes J, Broglio S (2017). Consensus statement on concussion in sport-the 5(th) international conference on concussion in sport held in Berlin, October 2016. Br J Sports Med.

[14] Cournoyer J, Hoshizaki TB (2019). Abnormal motor response associated with concussive injuries: biomechanical comparison between impact seizures and loss of consciousness. J Athl Train.

[15] Browning RA (1985). Role of the brain-stem reticular formation in tonic-clonic seizures: lesion and pharmacological studies. Fed Proc.

[16] Kreindler A, Zuckermann E, Steriade M, Chimion D (1958). Electro-clinical features of convulsions induced by stimulation of brain stem. J Neurophysiol.

[17] Aubry M, Cantu R, Dvorak J, Graf-Baumann T, Johnston K, Kelly J (2002). Summary and agreement statement of the first international conference on concussion in sport, vienna 2001. Phys Sportsmed.

[18] McCrory P, Johnston K, Meeuwisse W, Aubry M, Cantu R, Dvorak J (2005). Summary and agreement statement of the second international conference on concussion in sport, prague 2004. Phys Sportsmed.

[19] McCrory P, Meeuwisse W, Johnston K, Dvorak J, Aubry M, Molloy M, et al. Consensus statement on concussion in sport-the 3rd International Conference on concussion in sport, held in Zurich, November 2008. J Clin Neurosc: Official J Neurosurg Soc Austral. 2009;16(6):755–63. 10.1016/j.jocn.2009.02.002 19410148

